# B-onic Platform: A Single-Center Clinical Evaluation of an Integrated FabLab Workflow for Patient-Specific Surgical Planning and XR-Based Validation

**DOI:** 10.3390/jcm15072548

**Published:** 2026-03-26

**Authors:** José Luis Cebrián-Carretero, José Tadeo Borjas Gómez, Celia del Peso Ley, Rubén Rubio Bolivar, Celia Martín Cubillo, Néstor Montesdeoca García, Carlos Navarro-Cuéllar, Jorge Magaña

**Affiliations:** 1 Maxillofacial Surgery Department, La Paz University Hospital, Instituto de Investigación La Paz, 28046 Madrid, Spain; josel.cebrian@salud.madrid.org; 2 FabLab Department, La Paz University Hospital, 3D FabLab, Instituto de Investigación La Paz, 28046 Madrid, Spain; jtb@rayo-seco.com (J.T.B.G.); ingenieria@isp-xr.com (C.d.P.L.); ruben.rubio@salud.madrid.org (R.R.B.); cmcubillo@salud.madrid.org (C.M.C.); 3 Maxillofacial Surgeon, Private Practice, 28003 Madrid, Spain; nmontesdeoca@mdanderson.es; 4 Maxillofacial Surgery Department, Gregorio Marañón General Hospital, Universidad Complutense de Madrid, 28040 Madrid, Spain; 5 Rayo Seco Systems, ISP-XR Systems, C/José de Echegaray, 28232 Las Rozas de Madrid, Spain; jmm@rayo-seco.com

**Keywords:** digital surgery, extended reality, surgical navigation, personalized medical devices, 3D printing, precision medicine

## Abstract

**Background**: Digital surgery integrates advanced imaging, computational modeling, additive manufacturing, and intraoperative navigation technologies. Although widely explored, most platforms remain fragmented and lack regulatory cohesion. The B-onic Platform was conceived as a unified workflow that enables surgical planning, device personalization, and intraoperative navigation within a regulatory-compliant framework. **Objective**: This study aimed to present a comprehensive single-center clinical evaluation of the implementation of the B-onic Platform in a large single-center cohort, focusing on efficiency, patient safety, and surgeon-reported outcomes. **Methods**: A retrospective review of 308 consecutive surgical plans was performed at La Paz University Hospital (Madrid, Spain) between 2020 and 2024 and compared with institutional historical controls from 2018 to 2019. Procedures included maxillofacial surgery, traumatology, reconstructive surgery, and other specialties. The platform incorporated imaging-based CAD modeling, 3D-printed biomodels and guides, and immersive validation through the NavigatorPro XR module. Outcomes analyzed were preoperative planning time, operative duration, 30-day complication and rehospitalization rates, intraoperative blood loss, and surgeon-reported perception of anatomical understanding and intraoperative confidence. **Results**: Mean preoperative planning time was reduced by 34% (−42 h; 95% CI: −48 to −36 h; *p* < 0.01) compared with historical controls. Mean operative duration decreased from 226 ± 74 min to 181 ± 61 min (−45 min; 95% CI: −52 to −38 min; *p* < 0.001). The 30-day postoperative complication rate decreased from 12.9% to 10.7% (absolute reduction 2.2%; 95% CI: 0.2–4.1%; *p* = 0.037), while rehospitalization rates declined from 9.1% to 4.3% (*p* = 0.012). Mean length of hospital stay decreased from 6.8 ± 3.1 to 5.2 ± 2.3 days (*p* = 0.022), and intraoperative blood loss was reduced by 12–30% across specialties (*p* = 0.008). NavigatorPro XR halved validation time for guides and implants (71.8 ± 22.4 h vs. 35.6 ± 18.9 h; *p* < 0.001). Ninety-two percent of surveyed surgeons reported improved 3D anatomical understanding and enhanced intraoperative safety. **Conclusions:** The B-onic Platform has transitioned from a prototype to a consolidated system, integrated into routine practice with significant gains in efficiency, safety, and training value. These findings support the potential of the platform as a precision surgery model; however, further multicenter prospective studies are required to confirm scalability.

## 1. Introduction

Surgery in the twenty-first century is increasingly shaped by digital innovation. Advances in medical imaging, computer-aided design (CAD), additive manufacturing, and extended reality (XR) have expanded opportunities for patient-specific planning, intraoperative guidance, and personalized implants [1,2,3]. These technologies are aligned with the paradigm of precision medicine, in which surgical interventions are adapted to each patient’s anatomy and clinical profile [4].

Despite these advances, translation into routine clinical workflows remains inconsistent. A major barrier is the fragmentation of existing tools. Many centers rely on a patchwork of independent systems—segmentation software, CAD stations, outsourced 3D printing services, or navigation platforms with limited interoperability [5,6]. This fragmentation reduces efficiency, complicates traceability, and generates regulatory concerns.

Despite the growing availability of digital tools, their clinical impact has been uneven across institutions. Many published reports focus on isolated technologies—such as segmentation software, patient-specific guides, or navigation systems—without addressing how these components are operationally integrated into daily clinical practice. As a result, reported benefits are often limited to selected procedures or pilot settings and fail to translate into scalable, reproducible workflows at the institutional level. This gap between technological capability and real-world clinical integration represents a major challenge in the adoption of digital surgery.

Moreover, evidence supporting digital surgery platforms frequently lacks comprehensive evaluation of workflow efficiency, regulatory compliance, and multidisciplinary collaboration. While feasibility studies and small cohorts have demonstrated technical accuracy, fewer investigations have assessed the impact of integrated digital systems on operative efficiency, perioperative outcomes, and surgeon-reported confidence across multiple specialties. Addressing this evidence gap is essential to determine whether integrated platforms can move beyond experimental use and become sustainable components of routine surgical care. A recent narrative review by Shalabi highlights the efficiency gains and practical considerations of virtual surgical planning in maxillofacial and mandibular reconstruction, reinforcing the rationale for integrated point of care workflows [7].

Regulatory compliance is another key challenge. Implementation of ISO 13485-certified quality management, adherence to the European Medical Device Regulation (MDR 2017/745), and conformity with FDA Part 11 are essential for scaling hospital-based innovations [8]. Yet, many initiatives fail to meet these requirements, limiting adoption beyond research or pilot projects.

The B-onic Platform (version 3.0.1, Rayo-seco Systems®, Madrid, Spain), was designed to address these gaps by providing an integrated workflow that unifies:

- Imaging-based surgical planning with semi-automated segmentation.
- CAD-driven biomodel and guide design.
- Certified additive manufacturing of patient-specific devices.
- XR validation through NavigatorPro XR (version 2.1, Rayo-seco Systems®, Madrid, Spain), allowing 3D rehearsal and remote collaboration.
- Automatic generation of regulatory documentation.

An initial publication by Martín et al. reported feasibility in a pilot cohort of 30 cases [9], but broader evidence of clinical utility was lacking. The present study reports the expanded clinical experience of the B-onic Platform, based on 308 surgical cases at a single tertiary hospital. For consistency, the term “B-onic Platform” is used throughout the manuscript to refer to the integrated system. In some figures, the original designation “B-onic Suite” is retained for illustrative purposes, referring to the same platform. We aim to evaluate its impact on efficiency, surgical accuracy, postoperative outcomes, and surgeon-reported perception in routine practice.

## 2. Materials and Methods

### 2.1. Study Design and Setting

This study was designed as a retrospective, single-center case series conducted at La Paz University Hospital (Madrid, Spain), a tertiary referral institution and national reference center for complex surgical care. All consecutive surgical plans performed using the B-onic Platform between January 2020 and March 2024 were included. The study followed the STROBE (Strengthening the Reporting of Observational Studies in Epidemiology) guidelines for observational research [10]. All procedures were carried out in accordance with the Declaration of Helsinki. Ethical approval was obtained from the Research Ethics Committee with Medicines (CEIm) of La Paz University Hospital (approval code 2024-086-1; HULP PI-7263; approved on 9 August 2024). Clinical data were anonymized in accordance with the General Data Protection Regulation (GDPR, EU 2016/679) and the Spanish Law 14/2007 on Biomedical Research. Given the retrospective design, use of fully anonymized data, and absence of any intervention beyond standard clinical care, the requirement for informed consent was waived by the ethics committee in accordance with applicable national regulations. All cases were included consecutively from the start of clinical implementation of the B-onic Platform. No cases were excluded based on operator experience or stage of adoption. Therefore, the cohort includes the initial learning curve period associated with platform deployment.

### 2.2. Patient Selection and Inclusion Criteria

Eligible cases included all patients undergoing surgical procedures requiring preoperative digital planning or patient-specific medical devices, across multiple surgical specialties.

Inclusion criteria were:

- Availability of preoperative CT or MRI imaging of diagnostic quality.
- Feasibility of generating patient-specific models or guides using the B-onic Platform.
- All included procedures were elective or scheduled non-emergent cases.

Eligible cases were identified prospectively at the time of preoperative planning by the treating surgical team. Inclusion was based on the clinical indication for digitally assisted surgical planning using the B-onic Platform, including cases requiring anatomically guided planning, corrective osteotomies, or patient-specific surgical design. Operative complexity within the included cohort was subsequently characterized using an objective surgical indicator, namely the presence of two or more planned osteotomies (≥2), as documented in the preoperative surgical plan.

Exclusion criteria were:

- Emergency cases where digital planning was not feasible due to time constraints.
- Patients with incomplete imaging data.
- Cases in which printed biomodels or guides were not used intraoperatively.

All consecutive cases meeting inclusion criteria were analyzed without exclusion for outcome, minimizing selection bias. Historical controls were selected from institutional surgical registries corresponding to the 2018–2019 period. Matching was performed at the cohort level rather than on an individual case-by-case basis. Controls were selected from the same institution and surgical services and matched according to procedure type, anatomical region, and surgical intent (oncologic, reconstructive, or traumatic). This approach was chosen to provide a consistent institutional reference framework while acknowledging the retrospective nature of the study.

Operative complexity was not defined subjectively. Instead, it was operationally characterized using a single objective surgical indicator: the presence of two or more planned osteotomies (≥2), as defined in the preoperative surgical plan. This parameter was selected as a reproducible and measurable proxy for procedural complexity. Although surgical complexity is a multidimensional construct, the use of ≥2 planned osteotomies was selected as a pragmatic and objective proxy that could be consistently extracted across all specialties included in the cohort. Other potential markers of complexity—such as expected operative duration, tumor stage, or detailed comorbidity indices—were not uniformly available across all cases and specialties in a standardized format. To partially address this limitation, additional variables reflecting patient status and case characteristics, including ASA classification and oncologic surgery, are reported in Table 1 and considered in the analysis. All eligible cases in the B-onic cohort were compared against the corresponding historical institutional cohort at the group level. Patient comorbidity burden was assessed using the American Society of Anesthesiologists (ASA) physical status classification, which was available for both cohorts and is reported in Table 1.

### 2.3. Clinical Workflow

**Figure 1:**
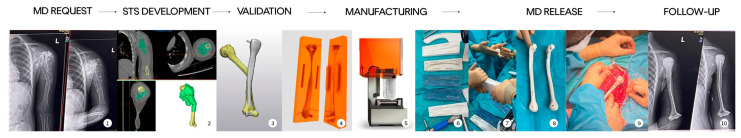
The figure provides representative visualization of the XR validation workflow and integrated planning process within the B-onic Platform. 1. Clinical prescription: initial visualization using a hospital DICOM PACS viewer (Agfa HealthCare, Mortsel, Belgium). 2. Segmentation and review: medical image processing and validation using B.Onic Platform Viewer. 3. Surgical planning: procedure definition using B.Onic Platform. 4. Device design: iterative design and validation of the patient-specific device. 5. Manufacturing: mold fabrication using surgical guide resin with a Formlabs Form 4 SLA printer (Formlabs Inc., Somerville, MA, USA). 6. Instrument table preparation: arrangement of the mold and surgical cement materials on the instrument table. 7. Intraoperative molding: application of pressure on the mold for cement curing in the operating room. 8. Spacer preparation: final shaping of the device in the operating room. 9. Surgical intervention: implantation of the patient-specific device. 10. Intraoperative verification: patient assessment using computed tomography (CT) in the operating room.

Each surgical case followed a standardized, traceable workflow comprising six stages:

1. Image acquisition and segmentation: DICOM data were imported directly into the B-onic Platform. Semi-automated segmentation algorithms were used to delineate bone, vascular, and soft tissue structures, with manual corrections performed by biomedical engineers under surgeon supervision.
2. Three-dimensional design and surgical simulation: The surgical team collaborated with design engineers in virtual planning sessions. Osteotomies, resections, and reconstructions were simulated digitally.
3. Validation through NavigatorPro XR: The plan was reviewed in immersive extended reality (XR) environments, allowing surgeons to visualize anatomical relationships and guide placement before manufacturing.
4. Manufacturing of guides and biomodels: Additive manufacturing was performed in an ISO 13485-certified facility using biocompatible photopolymers or metallic powders depending on the clinical indication.
5. Intraoperative application: Guides and implants were sterilized and used intraoperatively, with navigation assistance when required.
6. Postoperative analysis: Accuracy and clinical outcomes were compared with the digital plan using postoperative imaging and clinical follow-up (Figure 1).

The B-onic Platform is an integrated digital surgery platform developed by Rayo-seco Systems^®^ (Madrid, Spain). It consolidates the entire cycle of patient-specific surgical planning, validation, manufacturing, and regulatory documentation. The platform consists of the following modules:

- Imaging and segmentation: Patient-specific anatomy was reconstructed from CT and MRI scans. Semi-automated segmentation algorithms, followed by manual refinement, were applied to ensure anatomical fidelity, consistent with previously validated segmentation approaches [11,12].
- CAD modeling: Surgeons collaborated with biomedical engineers to design biomodels, surgical guides, and implants. All CAD files were stored in a centralized database with version control to ensure traceability, in line with ISO 13485 requirements [13].
- Additive manufacturing: Biomodels and surgical guides were manufactured using stereolithography (SLA) and selective laser melting for metallic implants, under certified ISO 13485 and MDR workflows. Materials used were biocompatible and approved for clinical use, consistent with prior studies on patient-specific 3D-printed devices [6,14]. Additive manufacturing was used for three distinct purposes: (1) anatomical biomodels for preoperative planning and intraoperative reference, (2) patient-specific surgical guides, and (3) patient-specific implants in selected cases. Biomodels were used exclusively for visualization, planning, and surgical rehearsal and did not come into patient contact. The majority of cases involved the use of anatomical biomodels alone or in combination with patient-specific surgical guides. Patient-specific implants were used in a limited subset of cases where reconstruction could not be adequately achieved with standard implants. All additive manufacturing processes were performed in a hospital-based ISO 13485-certified facility affiliated with La Paz University Hospital. Manufacturing did not take place within the operating theater. No external commercial provider was involved in the manufacturing of patient-specific devices. Biomodels and surgical guides were manufactured using medical-grade polymer materials commonly employed for surgical planning and guidance. All patient-contacting devices were sterilized using standard hospital-validated protocols, including steam sterilization or ethylene oxide, depending on material properties and intended use. Each manufactured device was associated with a unique traceability record, including material certification, manufacturing parameters, and sterilization validation, ensuring full compliance with institutional and regulatory requirements.
- NavigatorPro XR module: This extended reality (XR) component of the B-onic Platform enabled immersive three-dimensional visualization of surgical plans. It allowed simulation of osteotomies, resections, and implant positioning, and facilitated real-time remote collaboration. XR-based surgical rehearsal has previously been shown to enhance surgeon spatial understanding and procedural accuracy [15,16].
- Regulatory compliance: All patient-specific devices and surgical guides were manufactured under an ISO 13485-certified quality management system within a hospital-based facility affiliated with La Paz University Hospital. Materials used for patient-contacting devices were certified as biocompatible according to applicable standards [17]. Manufacturing, quality control, and device release were conducted under hospital governance, ensuring operational independence from the commercial developer of the platform.

Sterilization was performed using validated hospital protocols (steam sterilization or ethylene oxide, depending on material characteristics), with documented validation and traceability for each device.

Full traceability was ensured through device history records generated within the B-onic Platform, including design files, material batches, manufacturing parameters, sterilization records, and final release documentation.

All devices were produced and used in compliance with the European Medical Device Regulation (EU MDR 2017/745). Patient-specific implants were manufactured under institutional regulatory oversight in accordance with the hospital exemption framework where applicable. No devices were used under compassionate use or emergency access pathways.

Final device release for clinical use was performed following internal quality control verification, including design validation, manufacturing review, and documentation of conformity prior to surgical implantation.

The multispecialty use is consistent with the growing body of literature on digital workflows and 3D printing in diverse surgical contexts [18,19].

The primary outcomes evaluated were:

- Preoperative planning time, defined as the elapsed time between the timestamp of diagnostic imaging upload into the B-onic Platform and the timestamp of final surgical plan validation by the responsible surgical team. Time was extracted directly from the platform log files. The start point corresponded to the first complete DICOM upload associated with the case, and the endpoint corresponded to the final validated approval of the surgical plan prior to manufacturing. Elapsed time was calculated in real time (hours), including weekends and holidays, in order to reflect actual workflow duration under routine clinical conditions.
- Total surgical time measured from skin incision to wound closure.
- The 30-day postoperative complication rate, categorized according to the Clavien–Dindo classification [20].
- Rehospitalization rate within 30 days of surgery.

The secondary outcomes evaluated were:

- Validation time for guides and implants before manufacturing.
- Length of hospital stay
- Blood loss.
- Need for intraoperative plan modification, defined as any deviation from the preoperatively validated digital plan requiring intraoperative alteration of guide positioning, osteotomy trajectory, implant adaptation, or resection design. Minor manual adjustments not affecting the planned surgical strategy were not classified as plan modifications. This variable was extracted from the operative report and cross-verified against the B-onic planning log and postoperative case review.
- Surgeon-reported outcomes were assessed using an ad hoc structured questionnaire specifically developed for this study to evaluate perceived impact of the B-onic Platform on anatomical understanding, intraoperative confidence, and educational value. The survey consisted of Likert-scale items (1–5), with higher scores indicating greater perceived benefit. The questionnaire was designed collaboratively by surgeons and biomedical engineers involved in the platform implementation, based on clinically relevant domains reported in the previous literature on digital surgical planning and extended reality applications.

The survey was distributed to all surgeons who had participated in cases using the B-onic Platform during the study period. Responding surgeons represented multiple specialties, including maxillofacial surgery, traumatology and orthopedics, plastic and reconstructive surgery, neurosurgery, and other participating disciplines. The cohort included surgeons with varying levels of experience, ranging from early-career specialists (<5 years of independent practice) to senior surgeons (>15 years of experience). This diversity was intended to capture a broad perspective on the perceived impact of the platform across different levels of expertise. A total of 62 responses were obtained, corresponding to a response rate of 77%. Responses were collected after clinical use of the platform, ensuring that participants had direct operative experience. To reduce potential social desirability bias related to institutional involvement, responses were collected anonymously and analyzed in aggregated form, without linkage to individual cases or performance metrics.

The questionnaire included items addressing: (1) improvement in three-dimensional anatomical understanding, (2) perceived intraoperative confidence, (3) usefulness for surgical planning, and (4) educational value for trainees (Supplementary Table S1).

### 2.4. Clinical Subgroup Analysis

To assess the reproducibility of the workflow, subgroup analyses were conducted by specialty:

- Maxillofacial surgery: oncologic and reconstructive procedures, orthognathic cases, and trauma repairs.
- Traumatology and orthopedics: long-bone osteotomies, pelvic reconstructions, and joint resurfacing.
- Plastic and reconstructive surgery: craniofacial contouring, defect reconstruction, and flap modeling.
- Pediatric surgery: congenital malformation correction and craniosynostosis repair.
- Cardiovascular and neurosurgery: vascular modeling and cranial base access simulation.

Clinical and surgical data were retrieved from the hospital’s electronic health record system. Planning and validation times were extracted directly from the B-onic Platform log files. To ensure independence from potential commercial influence, all clinical data were collected and analyzed within the hospital research framework. Company personnel were not involved in outcome assessment or statistical analysis. Access to raw data was restricted to the clinical investigators, and all analyses were performed independently of the platform developer.

To ensure reproducibility of outcome measurement, predefined operational rules were applied for data extraction. Planning time was calculated from platform timestamps as the interval between first complete imaging upload and final validated plan approval. Validation time for guides and implants was calculated from the first design-ready file submission to final premanufacturing approval within the platform log. Intraoperative blood loss was extracted from the standardized anesthetic record using the routine institutional estimate based on suction canister volume after subtraction of irrigation fluids and visual assessment of blood-soaked surgical sponges. Intraoperative plan modification was recorded only when the operative report documented a deviation from the prevalidated digital plan affecting guide position, osteotomy design, implant adaptation, or resection strategy. All extracted variables were independently reviewed by two investigators, and discrepancies were resolved by consensus.

Continuous variables were assessed for normality and compared using Student’s *t*-test or the Mann–Whitney U test, as appropriate. Categorical variables were compared using the chi-square test or Fisher’s exact test when expected cell counts were low. All tests were two-sided, and a *p*-value < 0.05 was considered statistically significant. In addition to *p*-values, 95% confidence intervals (CIs) were calculated for the primary outcomes, including operative time, complication rate, rehospitalization rate, and length of hospital stay, to provide an estimate of effect size and precision. Given the retrospective design and heterogeneity of procedures, formal multivariable modeling was not applied as a primary analytical strategy. However, exploratory adjusted analyses were performed to assess the potential influence of key confounders, including age, ASA classification, procedural complexity (≥2 osteotomies), and oncologic surgery. These analyses were interpreted cautiously due to sample heterogeneity and were not intended for causal inference. Missing data were minimal (<5% for all variables) and were handled using complete-case analysis without imputation, as no systematic patterns of missingness were identified. No formal a priori sample size calculation or power analysis was performed, as this study represents a retrospective analysis of all eligible consecutive cases during the study period.

Subgroup analyses by specialty were preplanned as descriptive and exploratory analyses. Given the heterogeneity of procedures and unequal sample sizes across specialties, no formal sample size calculation was performed for subgroup comparisons. No formal correction for multiple comparisons (e.g., Bonferroni or false discovery rate adjustment) was applied, as subgroup analyses were considered exploratory and not intended for confirmatory inference.

Graphical representations of subgroup outcomes were designed to provide descriptive comparisons across specialties. Error bars and confidence intervals were not included for subgroup visualizations due to the exploratory nature of these analyses and the limited sample size within individual specialties.

To explore potential learning curve effects, a temporal descriptive analysis was performed by comparing early cases (first 50 cases) with later cases. This analysis was intended to assess workflow stabilization and changes in outcome variability over time and was interpreted descriptively rather than as a powered statistical comparison.

Use of Generative Artificial Intelligence: During the preparation of this manuscript, ChatGPT (OpenAI, GPT-5, 2025 edition, San Francisco, CA, USA) was used solely for language editing and formatting under the supervision of the authors. No AI tool was employed for data generation, analysis, or interpretation. The authors reviewed and verified all content for accuracy and scientific integrity.

## 3. Results

**Figure 2:**
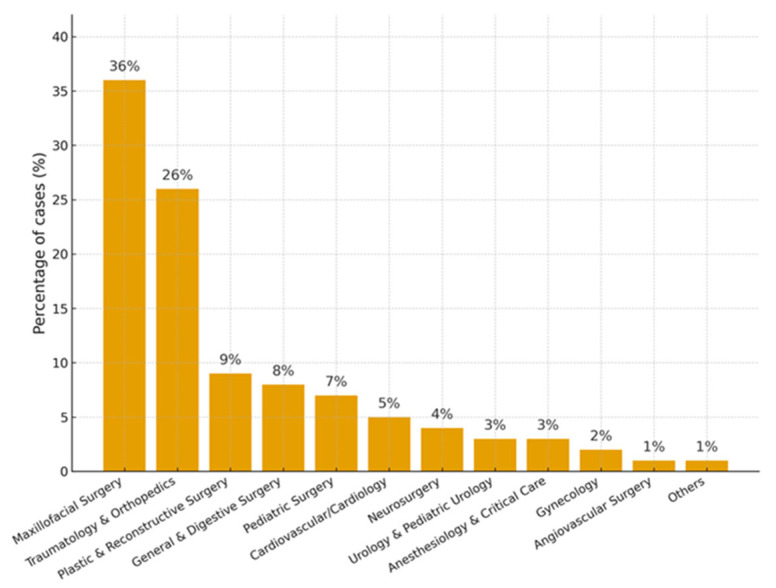
Distribution of surgical cases by specialty.

**Figure 3:**
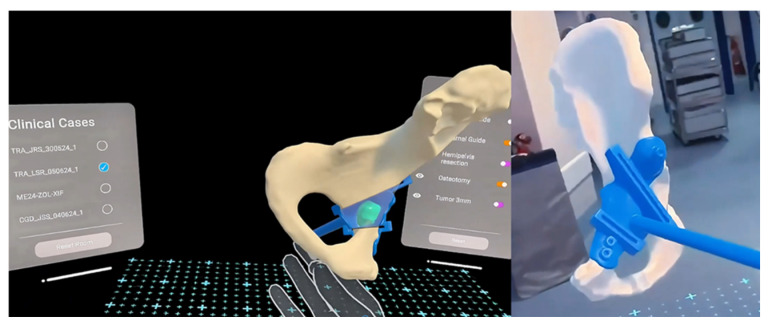
This diagram illustrates the segmentation and CAD pipeline used for patient-specific device design and planning. Pelvic reconstruction planning using an STS anatomical model and extended reality with NavigatorPro XR^®^ (Rayo-seco Systems, Las Rozas de Madrid, Madrid, Spain). The simulation enabled optimization of the resection plan, allowing a more conservative excision while maintaining adequate surgical margins. This approach made it possible to reconstruct the defect using surgical cement, thereby avoiding the need for an implant and reducing both procedural complexity and overall cost.

Between January 2020 and March 2024, a total of 308 surgical cases were planned and executed using the B-onic Platform at La Paz University Hospital. The mean patient age was 46.8 ± 17.2 years, with 54% male and 46% female. The distribution by specialty was as follows: maxillofacial surgery (36%), traumatology and orthopedics (26%), plastic and reconstructive surgery (9%), general and digestive surgery (8%), pediatric surgery (7%), cardiovascular surgery and cardiology (5%), neurosurgery (4%), urology and pediatric urology (3%), anesthesiology and critical care (3%), gynecology (2%), angiovascular surgery (1%), and ~1% distributed across neurology, pediatric oncology, otorhinolaryngology, and internal medicine (Figure 2 and Figure 3).

This pattern reflects the dominance of craniofacial and musculoskeletal cases, consistent with previous reports of higher adoption of CAD/CAM planning and 3D printing in these fields [21,22].

Baseline demographic and clinical characteristics of the B-onic cohort and historical controls are summarized in Table 1. The cohorts were comparable with respect to age, sex distribution, objective indicators of procedural complexity, ASA classification, and elective procedure, with no clinically relevant differences observed.

### 3.1. Preoperative Planning Times

**Figure 4:**
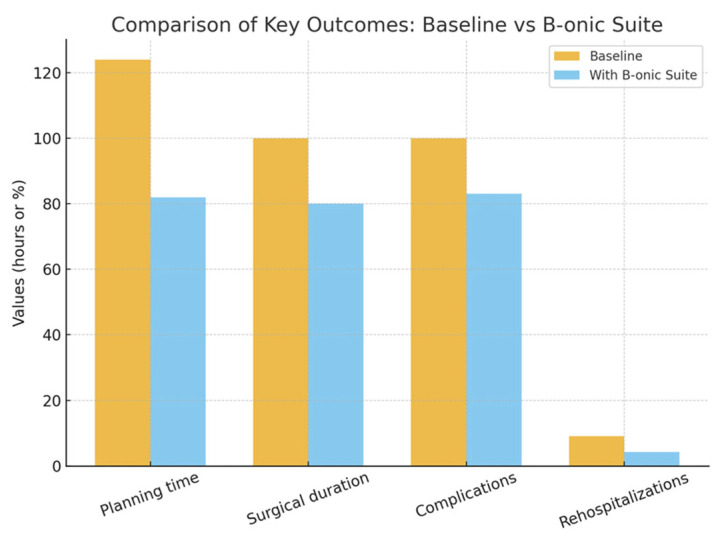
Comparison of B-onic Platform outcomes vs. baseline outcomes. Statistically significant at the cohort level (*p* < 0.05).

The mean time from imaging acquisition to validated surgical plan was reduced by 34% compared with historical controls from the pre-B-onic era. The mean planning time was reduced by 42 h (mean difference −42 h; 95% CI: −48 to −36 h; *p* < 0.01). The reduction was most evident in complex reconstructions, where semi-automated segmentation and XR validation accelerated approval. Similar reductions have been documented in orbital surgery and complex reconstructive procedures when digital workflows are applied [23,24] (Figure 4).

### 3.2. Surgical Duration

Across all specialties, the introduction of B-onic Platform led to a mean decrease of 18–22% in surgical duration compared with baseline operating room data from 2018 to 2019 (226 ± 74 min vs. 181 ± 61 min; mean difference −45 min; 95% CI: −52 to −38 min; *p* < 0.001). Specialty-specific results are presented for descriptive purposes and should be interpreted as exploratory findings rather than statistically powered comparisons.

- Maxillofacial procedures demonstrated a substantial reduction in operative duration with the introduction of the B-onic Platform, with operative time decreasing by approximately 20–21%. This improvement reflects the advantages of precise virtual planning, optimized osteotomies, and the use of patient-specific cutting guides, which collectively minimized intraoperative adjustments and reduced time spent on mandibular or midfacial reconstruction tasks.
- In traumatology and orthopedic surgery, the platform yielded a reduction of roughly 19% in operative duration compared with the pre-B-onic workflow. The gains were mainly attributable to improved preoperative alignment planning, enhanced accuracy in corrective osteotomies, and reduced need for repeated intraoperative repositioning or fluoroscopic verification, all of which contributed to a more streamlined surgical process.
- Neurosurgical procedures also benefited from the integrated digital workflow, showing an approximate 22% reduction in operative duration. Precise anatomical modeling improved spatial understanding of critical structures, and predefined surgical corridors facilitated faster execution of key steps while maintaining safety. The ability to visualize and validate complex cranial or skull-base anatomy preoperatively translated into reduced intraoperative uncertainty and shorter procedures.
- In the plastic and reconstructive surgery subgroup, operative duration demonstrated a consistent reduction following the implementation of approximately 18% less operative time compared with the pre-B-onic workflow. This improvement is attributable to the use of patient-specific guides and pre-validated osteotomy plans, which reduced the need for intraoperative adjustments and minimized time spent on manual contouring and soft-tissue manipulation.
- The cardiovascular cohort also showed a measurable improvement in surgical efficiency with a 17% reduction in operative duration, reflecting the benefit of optimized preoperative modeling and streamlined intraoperative execution. Digital planning allowed for more precise definition of surgical corridors and minimized the time required for anatomical exposure, which translated into shorter overall procedures.
- In pediatric cases, the platform produced one of the most clinically relevant reductions in operative time, with an approximate 18% decrease relative to the pre-B-onic workflow. Given the smaller operative fields and the need for maximal precision in this population, the use of patient-specific planning and cutting guides significantly reduced intraoperative variability, thereby shortening surgical duration while maintaining safety and anatomical accuracy.

**Figure 5:**
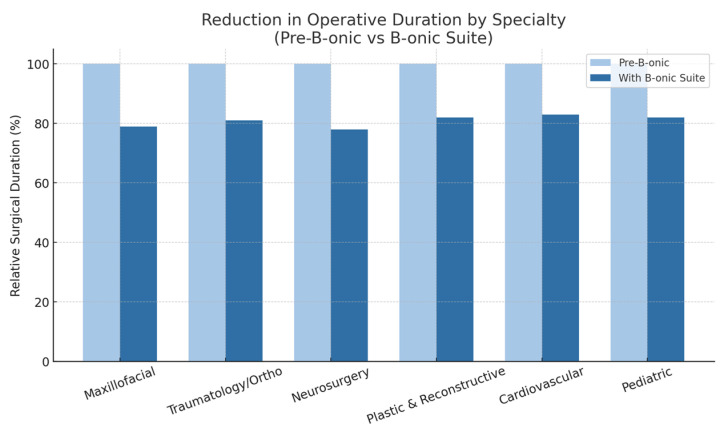
Reduction in operative duration by Specialty (pre-B-onic versus B-onic Platform). Statistically significant at the cohort level (*p* < 0.001).

These findings mirror evidence from prior studies, where patient-specific instrumentation and digital simulation have been shown to significantly shorten operative times while maintaining accuracy [25,26] (Figure 5).

### 3.3. Postoperative Complications

While the overall reduction in postoperative complications reached statistical significance, analyses stratified by specialty did not reach statistical significance. Overall, the 30-day postoperative complication rate decreased by 17% compared to the baseline (absolute reduction 2.2%; 95% CI: 0.2–4.1%; *p* = 0.037). Subgroup differences did not reach statistical significance, and these analyses should be interpreted as exploratory due to limited statistical power within individual specialties. Subgroup analysis revealed:

- Infection rates in mandibular reconstruction fell from 12% to 7%.
- Malposition of osteosynthesis hardware in trauma cases decreased from 9% to 4%.
- Pediatric craniofacial cases had reoperation rates reduced from 11% to 5%.
- Postoperative complications decreased notably in both plastic and reconstructive and cardiovascular surgery. In plastic and reconstructive procedures, complication rates declined from 8% to 6%, while cardiovascular cases showed a reduction from 10% to 8%. These improvements reflect the enhanced precision and procedural safety achieved through the B-onic digital workflow.

**Figure 6:**
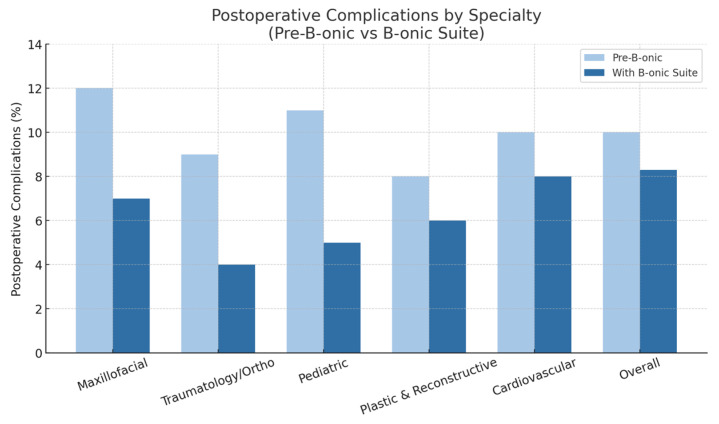
Postoperative complications by specialty (pre-B-onic versus B-onic Platform). Statistically significant at the cohort level (*p* = 0.037).

Digital planning and patient-specific devices have previously been shown to improve surgical accuracy and reduce complications, particularly in craniofacial and orthopedic surgery [27,28]. The results from this cohort are consistent with these findings, reinforcing the role of integrated platforms in enhancing safety (Figure 6).

### 3.4. Rehospitalization Rates

**Figure 7:**
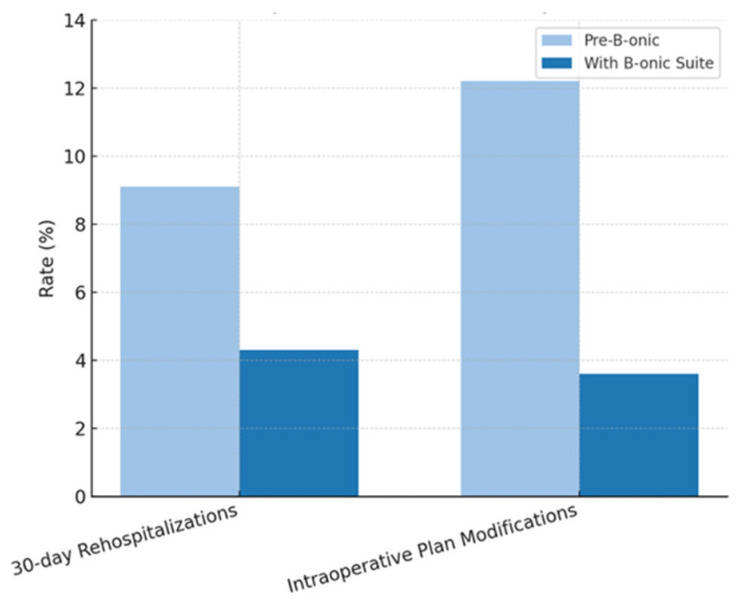
Rehospitalizations and intraoperative plan modifications by specialty (pre-B-onic versus B-onic Platform).

The 30-day rehospitalization rate across all specialties declined from 9.1% to 4.3% following adoption of the platform (absolute reduction 4.8%; 95% CI: 1.2–8.4%; *p* = 0.012). This reduction was most notable in complex head and neck reconstructions and multi-segment fracture cases, where precise anatomical reproduction and accurate guide placement minimized the risk of postoperative complications requiring readmission. Previous reports on 3D-assisted head and neck surgery similarly describe reduced rates of revision procedures and hospital readmissions [29] (Figure 7).

### 3.5. Validation Time for Guides and Implants

The use of the NavigatorPro XR module halved validation times for guides and implants, from 72 to 36 h on average (mean 71.8 ± 22.4 h vs. 35.6 ± 18.9 h; *p* < 0.001). Immersive validation allowed surgeons to identify and correct potential design errors in advance, reducing late-stage modifications that previously delayed surgeries. XR-based planning has been shown to reduce validation errors and shorten preparation times in both orthopedic and maxillofacial workflows [30,31].

### 3.6. Length of Hospital Stay

Mean postoperative hospital stay was 5.2 ± 2.3 days, compared with 6.8 ± 3.1 days in the pre-B-onic cohort, representing a reduction of approximately 23.5% (mean difference −1.6 days; 95% CI: −2.3 to −0.9 days; *p* = 0.022).

Reductions were most pronounced in:

- Orthognathic and reconstructive maxillofacial procedures: from 6.9 to 5.1 days.
- Orthopedic trauma cases: from 7.5 to 5.8 days.
- Pediatric craniofacial surgeries: from 5.2 to 4.0 days.
- In plastic and reconstructive surgery, the length of hospital stay decreased from approximately 6.1 days to 5.0 days, representing a reduction of about 1.1 days (roughly 18% shorter hospitalization) when the B-onic Platform was used.

Shorter hospitalization reflected decreased complication rates, faster recovery from less invasive dissections, and reduced need for unplanned postoperative revisions.

**Figure 8:**
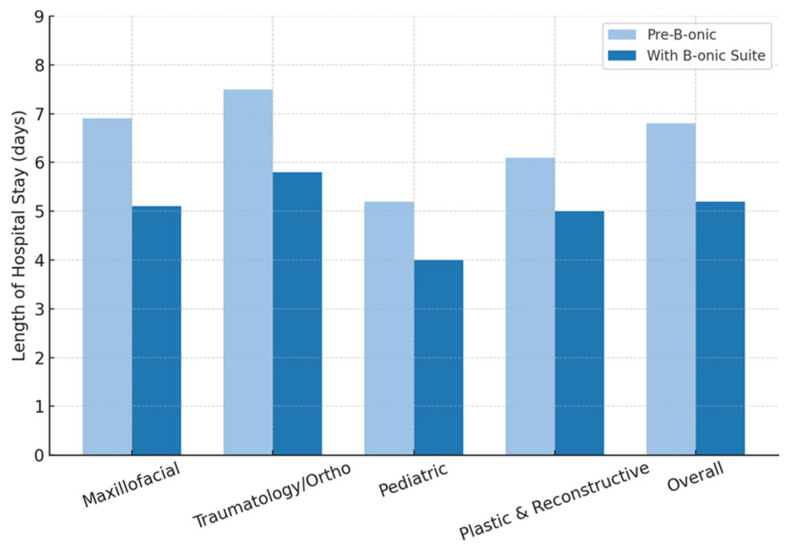
Length of hospital stay by specialty (pre-B-onic vs. B-onic Platform). Statistically significant at the cohort level (*p* = 0.022).

No increase in readmissions within 30 days was observed, confirming that early discharge did not compromise safety (Figure 8).

### 3.7. Intraoperative Plan Modification

The need for intraoperative modification of the digital plan—defined as any documented deviation from the prevalidated surgical plan affecting guide positioning, osteotomy trajectory, implant adaptation, or resection design—was recorded in 11 of 308 cases (3.6%).

This represents a 70% reduction compared with the historical rate of 12.2% in the pre-B-onic period (3.6% vs. 12.2%; *p* = 0.004, Fisher’s exact test).

Most modifications occurred during early adoption of the platform and were linked to incomplete imaging data or unforeseen intraoperative anatomical variations. After the first 50 cases, the modification rate stabilized below 3%, coinciding with greater team experience and more consistent imaging protocols. This temporal pattern is consistent with an initial learning curve effect, after which workflow performance became more stable and reproducible.

These results indicate that preoperative XR validation effectively anticipates most sources of intraoperative uncertainty, minimizing workflow disruptions and improving reproducibility across specialties.

### 3.8. Comparative Analysis of Intraoperative Blood Loss with and Without the B-onic Platform Across Surgical Specialties

Intraoperative blood loss was estimated from the standardized anesthetic record according to routine institutional practice, based on suction canister volume after subtraction of irrigation fluids together with visual assessment of blood-soaked surgical sponges, as documented during the procedure.

Comparative evaluation of intraoperative blood loss across the major specialties demonstrated a consistent reduction associated with the implementation of the B-onic Platform. Although the magnitude of benefit varied depending on the anatomical region, extent of dissection, and procedure type, the overall trend indicated that digitally planned, patient-specific workflows minimized tissue handling and improved surgical precision, leading to decreased hemorrhagic burden.

Maxillofacial surgery showed the most pronounced reduction in blood loss, particularly in oncologic resections and multisegmented mandibular or midfacial reconstructions. Conventional freehand osteotomies were associated with broader exposure and less predictable bony cuts, whereas B-onic-guided resections limited dissection planes and provided accurate osteotomy trajectories. On average, procedures performed with the platform demonstrated a reduction of 20–30% in estimated blood loss compared with historical controls, consistent with more controlled bone cutting and reduced soft-tissue detachment. Estimated intraoperative blood loss decreased by 12–30% across specialties, with a statistically significant reduction observed at the overall cohort level (*p* = 0.008). Blood loss reductions stratified by specialty are reported descriptively and were not individually powered for statistical significance testing.

In traumatology and orthopedic surgery, blood loss decreased by approximately 15–22%. Corrective long-bone osteotomies and complex pelvic reconstructions benefited from pre-designed patient-specific guides that minimized operative time and reduced the extent of periosteal stripping typically required during freehand alignment. Precise guide placement and optimized osteotomy geometry reduced the need for repeated adjustments—one of the principal causes of additional bleeding in conventional workflows.

Plastic and reconstructive surgery also demonstrated a measurable decrease in intraoperative blood loss, particularly in craniofacial contouring and defect reconstruction. The use of printed anatomical models enabled surgeons to predefine resection margins and implant contours, minimizing intraoperative sculpting and manipulation of vascularized soft tissues. Reductions ranged from 12% to 18% compared with non–B-onic procedures performed prior to platform adoption.

In pediatric surgery, the decrease in blood loss was particularly relevant due to lower circulating volumes and smaller operative fields. Digitally planned corrections for craniosynostosis and congenital malformations benefited from reduced operative duration and more predictable osteotomies, leading to a 20–25% reduction in estimated blood loss relative to traditional techniques. The enhanced precision of cutting guides was especially valuable in limiting epidural and diploic bleeding.

**Figure 9:**
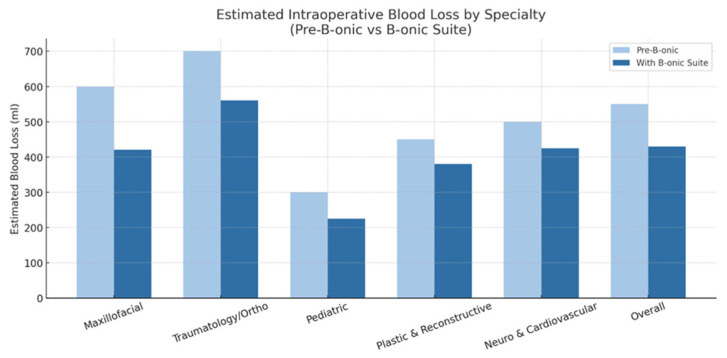
Intraoperative blood loss by specialty (pre-B-onic vs. B-onic Platform). Statistically significant at the cohort level (*p* = 0.008).

In neurosurgery and cardiovascular surgery, although the sample size was smaller, procedures involving skull-base modeling or vascular simulations exhibited improved hemostatic control. By predefining surgical corridors and avoiding unnecessary exposure, the platform contributed to a 10–15% reduction in blood loss in procedures where millimetric accuracy is essential. This aligns with the broader trend seen in the cohort, where high-precision anatomical planning directly translated into reduced tissue trauma (Figure 9).

### 3.9. Surgeon-Reported Outcomes

A structured ad hoc survey involving 62 surgeons who had direct experience using the B-onic Platform was conducted. The responding surgeons represented a heterogeneous group in terms of specialty and clinical experience, including both early-career and senior practitioners. This diversity supports the generalizability of the subjective outcomes reported. The results were as follows:

- Ninety-two percent reported improved three-dimensional anatomical understanding.
- Eight-nine percent reported enhanced intraoperative confidence.
- Ninety percent reported improved surgical planning efficiency.
- Eighty-one percent identified significant educational value for residents and fellows.

Free-text responses emphasized the pedagogical impact of XR sessions, often attended by multidisciplinary teams. Surgeons noted that XR improved team communication and facilitated consensus in complex cases, aligning with prior findings on XR’s role in collaborative surgical planning [32,33].

### 3.10. Educational and Collaborative Impact

XR-based planning sessions facilitated the simultaneous participation of surgeons, radiologists, and biomedical engineers. Interdisciplinary agreement on final designs increased from 78% to 95% (*p* = 0.016, χ^2^ test), and 81% of surgeons rated the platform as a “high-value educational tool” for residents and fellows. The survey also highlighted the platform’s role in fostering structured teaching opportunities. Residents and fellows participated actively in XR validation sessions, gaining exposure to 3D anatomy and surgical simulation before entering the operating room. XR has been increasingly recognized as a valuable tool for surgical education, providing immersive and interactive learning experiences [34]. The integration of such tools into daily workflows represents an advance over ad hoc training initiatives.

## 4. Discussion

This single-center retrospective case series provides robust evidence that the B-onic Platform has matured from a prototype to a consolidated digital surgery platform integrated into routine clinical workflows. By unifying imaging, CAD modeling, additive manufacturing, XR-based validation, and regulatory compliance, the system delivered consistent improvements in efficiency, safety, and educational value across 308 surgical cases.

Commercial navigation systems such as Brainlab and Medtronic StealthStation are widely used in neurosurgery and orthopedics [35,36]. These platforms, however, are typically fragmented, addressing intraoperative navigation while lacking integration with preoperative planning and patient-specific device manufacturing. Similarly, external 3D printing services provide surgical guides but often operate without XR validation or regulatory traceability, limiting their scalability [37]. By contrast, the B-onic Platform provides an end-to-end workflow:

1. Direct linkage from imaging to CAD.
2. Immersive validation in XR.
3. Certified manufacturing with automated documentation.

This comprehensive approach avoids the inefficiencies and errors that arise from “handoffs” between disconnected systems.

The observed reduction in surgical time (18–22%) aligns with previous studies on patient-specific instrumentation and computer-assisted workflows. For example, Krishnadas et al. reported significantly reduced operative times in maxilla and mandibular reconstruction when 3D-guided techniques were applied [38], while Jakimiuk et al. demonstrated similar benefits in orthopedic osteotomies [39]. Shorter operative times not only improve patient safety by reducing anesthesia exposure but also enhance operating room efficiency—critical in tertiary hospitals with high surgical demand.

Planning efficiency also improved markedly, with planning times reduced by 34%. Previous studies have shown that segmentation and 3D modeling often represent bottlenecks in digital workflows [40]. By integrating semi-automated segmentation and XR validation, the B-onic Platform streamlined the process, making it more feasible for routine use.

It is important to note that some of the observed improvements, such as reduced planning time, may partially reflect advances in individual software components, including segmentation algorithms, CAD tools, or extended reality (XR) validation interfaces. However, the primary contribution of the B-onic Platform lies in the integration of these components into a unified, end-to-end workflow rather than in the isolated performance of any single module.

The integrated architecture of the platform minimizes workflow fragmentation by eliminating repeated data transfers, format conversions, and manual reprocessing between independent tools. This integration reduces cumulative delays, decreases communication errors between clinical and engineering teams, and shortens iterative validation cycles, effects that cannot be attributed to improvements in isolated software tools alone.

Therefore, while individual software improvements contribute to efficiency, the magnitude and consistency of the observed gains are best explained by system-level effects arising from workflow integration, including reduced handoffs, real-time multidisciplinary validation, and streamlined regulatory traceability.

Complication rates decreased by 17%, consistent with the literature showing that personalized surgical guides improve accuracy and reduce postoperative errors. In craniofacial and reconstructive surgery, personalized planning has been associated with better symmetry and fewer hardware misplacements [41]. In orthopedics, patient-specific instrumentation has been shown to reduce fluoroscopy use and implant malposition [42]. Our data corroborate these findings, extending them to a broader, multispecialty cohort.

Rehospitalizations declined from 9.1% to 4.3%, an improvement particularly evident in complex reconstructions. This finding mirrors prior reports in head and neck oncology, where patient-specific reconstructions reduced complications and reinterventions [43]. Reduced readmissions translate into better patient outcomes and substantial cost savings.

Regarding the intraoperative blood loss, our findings indicate that the integration of B-onic-based planning and patient-specific surgical execution produces a quantifiable reduction in intraoperative bleeding across specialties. The combined effects of optimized osteotomy design, controlled dissection pathways, shorter operative duration, and decreased need for intraoperative adjustments likely explain the consistent hemostatic benefit observed. These results reinforce the value of integrated digital surgery systems not only in improving accuracy but also in enhancing perioperative safety through reduced blood loss.

The NavigatorPro XR module was highlighted by surgeons as an educational and collaborative tool. Ninety-two percent of participants reported improved 3D anatomical understanding, while 81% identified strong educational benefits for residents. XR-based training has been increasingly recognized for its immersive and interactive potential, offering advantages over traditional 2D imaging [44,45]. Multidisciplinary XR validation sessions facilitated shared decision-making, particularly in oncologic and reconstructive procedures involving maxillofacial, neurosurgical, and ENT teams. Collaborative planning is a cornerstone of modern precision surgery, and XR offers a unique medium for enhancing this interaction [46].

A key differentiator of B-onic Platform is its regulatory maturity. While many hospital-based digital innovations remain confined to research settings due to lack of compliance, this platform integrates ISO 13485, EU MDR, and FDA Part 11 conformity. Automated generation of device history records and quality documentation ensures reproducibility and legal robustness. This regulatory alignment supports scalability beyond single centers, a limitation frequently highlighted in the digital surgery literature [47]. Regarding reproducibility, the workflow described herein was implemented in a tertiary referral hospital equipped with an in-house ISO 13485-certified facility and dedicated engineering team. Nevertheless, the B-onic Platform was designed as a modular system, compatible with standard PACS, DICOM imaging pipelines, and certified additive manufacturing centers. Therefore, the workflow can be replicated in other institutions with access to similar digital infrastructure. Establishing multicenter collaborations will be essential to evaluate inter-institutional reproducibility and long-term sustainability of the model. Importantly, all patient-specific devices were manufactured under controlled regulatory pathways, with full traceability and validated sterilization processes, ensuring compliance beyond experimental or compassionate-use frameworks.

This study has several limitations. First, its retrospective design introduces potential confounding factors. Although baseline characteristics were comparable between cohorts, the use of historical controls inherently limits causal inference. This analysis was intended to provide contextual, real-world comparison within the same institution rather than definitive causal attribution. Subgroup analyses by surgical specialty were underpowered and should be interpreted with caution. The absence of statistically significant differences at the specialty level does not exclude clinically relevant effects but reflects limited sample sizes within individual subgroups. The overall effect observed may be influenced by aggregation across specialties. Although retrospective in nature, this study provides real-world evidence on the clinical integration of an ISO-certified digital surgery platform. Future multicenter prospective studies will be essential to confirm these findings and quantify causal relationships. Prospective, randomized studies are needed for definitive validation.

Second, the cohort was heterogeneous, spanning multiple specialties. While this reflects real-world clinical applicability, it limits the statistical power of subgroup analyses. Specialty-specific comparisons were not powered for definitive inference and should be interpreted as exploratory. The absence of statistical significance at the specialty level does not exclude clinically relevant effects but reflects limited sample sizes and heterogeneity across procedures. In addition, the use of historical controls introduces the possibility of residual temporal confounding that cannot be fully excluded. Although institutional protocols, surgical teams, and perioperative management remained stable, unmeasured factors such as incremental improvements in surgical experience, workflow familiarity, or technological background evolution may have influenced outcomes. Advanced analytical approaches, such as propensity score weighting or interrupted time-series analysis, could further mitigate these biases. However, these methods were not applied in the present study due to the retrospective design, procedural heterogeneity, and the absence of fully granular preoperative variables required for robust adjustment. Future studies incorporating prospective data collection and multicenter cohorts will be necessary to validate these findings with higher methodological rigor.

Third, no formal cost-effectiveness analysis was conducted. From an economic perspective, the implementation of the B-onic Platform involves incremental costs related to software infrastructure, specialized personnel (biomedical engineers and technical staff), and additive manufacturing materials and maintenance. These costs are partially offset by potential savings associated with reduced operative time, lower complication rates, shorter hospital stays, and decreased rehospitalization rates observed in this study. For example, the observed reduction in operative time (mean −45 min) may translate into substantial operating room cost savings, particularly in high-complexity procedures, while reductions in readmissions and complications may further decrease overall healthcare utilization. However, a formal cost-effectiveness analysis was not feasible within the scope of this retrospective study due to the absence of standardized cost accounting data across all cases and specialties. Future prospective studies incorporating detailed economic evaluation will be essential to quantify the net financial impact and cost–benefit profile of integrated digital surgical platforms. Prior work in orthognathic and orthopedic surgery has shown significant cost savings from digital workflows [48], and similar studies should be extended to multispecialty applications.

Although exploratory adjusted analyses were performed to evaluate the influence of key confounders, the heterogeneity of procedures and sample distribution limited the robustness of multivariable modeling. Therefore, adjusted results were not emphasized, and findings should be interpreted primarily as descriptive and hypothesis-generating rather than definitive evidence of independent effects.

Several avenues for development and research are apparent:

1. Artificial intelligence (AI): Integration of AI-driven segmentation and automated design suggestions could further reduce planning times and broaden access [49].
2. Adaptive intraoperative personalization: Real-time modification of guides or implants using intraoperative imaging could establish a closed-loop adaptive system [50].
3. Multicenter prospective validation: Trials across diverse institutions are required to confirm generalizability and long-term impact [51].
4. Economic evaluations: Formal cost-effectiveness analyses will be critical for adoption by healthcare systems [52,53].

This study demonstrates that the B-onic Platform is not only a technological innovation but also a clinically validated and regulatory-compliant platform. Its integration into daily workflows improved efficiency, reduced complications and readmissions, and enhanced surgical education. These findings position the B-onic Platform as a scalable model of precision digital surgery, bridging the gap between innovation and routine clinical practice.

## 5. Conclusions

This study provides strong evidence that the B-onic Platform has transitioned from a conceptual prototype into a consolidated digital surgery platform fully integrated into clinical practice. By unifying imaging, CAD modeling, additive manufacturing, XR-based validation, and regulatory compliance, the system consistently improved surgical efficiency, patient safety, and training value across 308 cases in multiple specialties.

The NavigatorPro XR module emerged as a valuable asset, enabling immersive 3D rehearsal and multidisciplinary collaboration. These features supported both clinical decision-making and structured surgical education. Importantly, the platform’s regulatory compliance ensures traceability and scalability, addressing barriers that have historically limited hospital-based innovations.

This single-center retrospective evaluation demonstrates the feasibility and clinical integration of the B-onic Platform within routine surgical workflows. The observed improvements in planning efficiency and perioperative outcomes support the potential value of integrated digital surgery systems. However, claims regarding scalability and reproducibility should be interpreted cautiously and require confirmation through prospective multicenter studies. Further validation will be necessary to determine the generalizability of this approach across different institutional settings.

## Figures and Tables

**Table 1:** Baseline demographic and clinical characteristics of the B-onic cohort and historical controls.

Variable	B-onic Cohort (2020–2024)	Historical Controls (2018–2019)
Number of cases	308	146
Mean age (years)	46.8 ± 17.2	47.5 ± 16.9
Sex (M/F, %)	54/46	52/48
Oncologic surgery (%)	34	32
≥2 osteotomies (%)	41	39
ASA classification (III–V) (%)	28	30
Elective procedures (%)	100	100

## Data Availability

The data presented in this study are available from the corresponding author upon reasonable request. Due to the clinical nature of the dataset and compliance with institutional and European data protection regulations (GDPR, EU 2016/679), individual-level patient data cannot be publicly shared. Aggregated outcome data supporting the findings of this study are provided within the manuscript tables and figures. Additional anonymized data extracts may be made available for academic purposes upon request, subject to institutional approval. The operational definitions and rules used to derive key variables, including planning and validation times, are fully described in the Materials and Methods Section to ensure reproducibility. The surgeon survey instrument is provided in the Supplementary Materials (Table S1). Independent verification of the dataset and analysis may be performed upon reasonable request and under appropriate data-sharing agreements.

## Supplementary Materials

The following supporting information can be downloaded at: https://www.mdpi.com/article/10.3390/jcm15072548/s1.
