# Supplementary material for: B-onic Platform: A Single-Center Clinical Evaluation of an Integrated FabLab Workflow for Patient-Specific Surgical Planning and XR-Based Validation
Source: J Clin Med. 2026 Mar 26;15(7):2548. doi: 10.3390/jcm15072548 (PMC13073156; doi:10.3390/jcm15072548)
Supplement: Supplementary file 1 [file jcm-15-02548-s001.zip › jcm-4164194-supplementary.pdf]

Supplementary Table S1. Surgeon Survey Questionnaire

Item	Domain	Statement (Likert scale 1–5)
1	Anatomical understanding	The B-onic Platform improved my three-dimensional understanding of surgical anatomy.
2	Intraoperative confidence	The B-onic Platform increased my intraoperative confidence.
3	Planning efficiency	The B-onic Platform improved surgical planning efficiency.
4	Educational value	The B-onic Platform has educational value for residents and fellows.

Likert scale: 1 = strongly disagree; 2 = disagree; 3 = neutral; 4 = agree; 5 = strongly agree.
